# Multi-criteria decision analysis for setting priorities on HIV/AIDS interventions in Thailand

**DOI:** 10.1186/1478-4505-10-6

**Published:** 2012-02-17

**Authors:** Sitaporn Youngkong, Yot Teerawattananon, Sripen Tantivess, Rob Baltussen

**Affiliations:** 1 Health Intervention and Technology Assessment Program (HITAP), Ministry of Public Health, Nonthaburi, Thailand; 2 Nijmegen International Center for Health Systems research and Education (NICHE), Department of Primary and Community Care, Radboud University Nijmegen Medical Center, Nijmegen, The Netherlands

**Keywords:** Multi-criteria decision analysis, Priority setting, HIV/AIDS interventions, Discrete choice experiment

## Abstract

**Background:**

A wide range of preventive, treatment, and care programs for HIV/AIDS are currently available and some of them have been implemented in Thailand. Policy makers are now facing challenges on how the scarce resources for HIV/AIDS control can be spent more wisely. Although effectiveness and cost-effectiveness information is useful for guiding policy decisions, empirical evidence indicates the importance of other criteria, such as equity and the characteristics of the target population, also play important roles in priority setting. This study aims to experiment with the use of multi-criteria decision analysis (MCDA) to prioritise interventions in HIV/AIDS control in Thailand.

**Methods:**

We used MCDA to rank 40 HIV/AIDS interventions on the basis of the priority setting criteria put forward by three groups of stakeholders including policy makers, people living with HIV/AIDs (PLWHA), and village health volunteers (VHVs). MCDA incorporated an explicit component of deliberation to let stakeholders reflect on the rank ordering, and adapt where necessary.

**Results:**

Upon deliberation, policy makers expressed a preference for programs that target high risk groups such as men who have sex with men, injecting drug users, and female sex workers. The VHVs preferred interventions that target the youth or the general population, and gave lower priority to programs that target high risk groups. PLWHA gave all interventions the same priority. The rank order correlation between the priorities as expressed before and after deliberation was 37% among the policy makers and 46% among the VHVs.

**Conclusion:**

This study documented the feasibility of MCDA to prioritize HIV/AIDS interventions in Thailand, and has shown the usefulness of a deliberative process as an integrated component of MCDA. MCDA holds potential to contribute to a more transparent and accountable priority setting process, and further application of this approach in the prioritisation of health interventions is warranted.

## Background

Since HIV/AIDS has long been recognized as a leading cause of death and a high burden of disease in Thailand [1-3], a wide range of preventive, treatment, and care programs have been implemented to combat the disease. Recently, it was suggested that funding decisions on these programs are not taken in a systematic manner and that the resulting mix of interventions is not offering the best value for money [4]. Consequently, Thai policy makers now face the challenge of how the scarce resources available for HIV/AIDS control can be spent more wisely.

A range of studies are available to guide Thai policy makers to prioritise HIV/AIDS interventions. International estimates are available on the effectiveness and cost-effectiveness of HIV/AIDS interventions [5-7], and a recent document has systematically reviewed this information - in combination with national estimates - to provide informed priorities for HIV/AIDS control [4]. Yet the analysis falls short of including other criteria that may also play important roles in effective decision-making, such as ethical and social concerns. For example, the preference of society to pursue not only efficiency goals (that could result in prevention-oriented strategies for the general population) but also equity goals (that could, for example, result in treatment-oriented strategies for the severely ill) may have a large impact on the choice of programs [8-10]. This indicates the need for multiple criteria decision analysis (MCDA) to account for other criteria beyond effectiveness and cost-effectiveness in the decision-making process [11-14].

Although MCDA is used in only a few applications to guide the making of resource allocation decisions on health, it is routinely used in environmental, agricultural and marketing sciences to set intervention priorities [14]. In those disciplines, MCDA has evolved as a response to the observed inability of people to effectively analyze multiple streams of dissimilar information. The analysis establishes preferences between interventions by reference to an explicit set of criteria that the decision-making body has identified. A key component of every MCDA is the performance matrix that describes the performance of the interventions against each criterion. The performance matrix may be the final product of the analysis, allowing the decision makers to qualitatively rank the interventions. Such intuitive processing of the data can be quick and effective, but it may also lead to the use of unjustified assumptions, causing an incorrect ranking of options. In analytically more sophisticated MCDA techniques, the information in the basic matrix is usually converted into consistent numerical values. The key idea is to construct scales representing preferences for the consequences, to weigh the scales for their relative importance, and then to calculate weighted averages across the preference scales [14]. In recent applications of MCDA [15-19], it has been criticized for its quantitative nature - studies typically rank ordered interventions on the basis of weighted averages and, in this way, consider quantifiable criteria only. To date, some attempts to capture non-quantifiable criteria to support the deliberative process have been reported [20,21]. This confirms that MCDA should rather include a deliberative process or other qualitative tools to also consider non-quantifiable concerns [20,22-25] and foster well-balanced judgments on intervention priorities [26,27].

The primary aim of this study is to experiment with the use of MCDA, including the use of a deliberative process to prioritise interventions in HIV/AIDS control in Thailand. This research follows up on a recent study that employed discrete choice experiments (DCE) to identify and measure the relative importance of various quantifiable and non-quantifiable criteria for the priority setting of HIV/AIDS interventions in Thailand among various stakeholders [28].

## Methods

The MCDA in the present study includes three components. Firstly, we assessed the performance of interventions on the criteria as identified in the DCE (i.e. we constructed the performance matrix). Second, we ranked ordered interventions. Third, we engaged with the various stakeholders in a deliberative process to adapt the rank ordering where necessary. We also compared the rank order of interventions before and after the deliberative process. These components are discussed in turn.

### Constructing the performance matrix

As a starting point, we identified a broad set of 40 HIV/AIDS interventions that are already implemented, or eligible for implementation, in Thailand. We then constructed the performance matrix, i.e. we scored each of the selected HIV/AIDS interventions as a function of their performance on a set of criteria as identified in a recent DCE study [28]. This study identified the criteria to be relevant to the priority setting of HIV/AIDS control in Thailand through group discussions with each group of stakeholders including policy makers, people living with HIV/AIDS (PLWHA), and community members represented by village health volunteers (VHVs). The resulting criteria from the three group discussions were compared and finally those that were identified by two or more discussion groups were selected. These included: target groups of interventions (i.e. children, teenagers, adults, and high-risk adults); gender of target groups (i.e. female versus male); type of interventions (i.e. prevention, treatment of patients with HIV, and treatment of patients with AIDS); effectiveness (i.e. low versus high effectiveness); and quality of evidence (i.e. weak versus strong evidence). In the performance matrix, '0' denotes the absence of and '1' indicates the presence of a criterion level (see Additional file 1: Appendix 1). Information on target group, gender of target group and type of intervention was identified from each intervention itself, whereas the information on effectiveness of intervention and quality of evidence on effectiveness were based on the review conducted by Pattanaphesaj and Teerawattananon [4].

### Rank ordering of interventions

Subsequently, we estimated the probability of selection of an intervention by using the logistic regression model derived from the DCE study [28]:

Logit(P) = ln [P/(1-P)] = β_0 _+ β_1-3 _Target group + β_4-5 _Gender of target group + β_6-7 _Type of intervention + β_8 _Effectiveness + β_9 _Quality of evidence on effectiveness + ε

where P is the probability of an intervention being selected by the respondents, β_0 _is the constant term, β_i _(i = 1-9) are the coefficients of the model indicating the probability of selection relative to the reference criterion level, and ε is the unobservable error term. The regression coefficients for all criteria were obtained from each of the three groups of stakeholders - policy makers, PLWHA, and community members - represented by VHVs during the DCE survey (These are listed in Additional file 1: Appendix 2). Next, all interventions were ranked in order of their probability of selection.

### Deliberative process

In the deliberative process, group discussions were independently organized between July and August 2009 with three groups of stakeholders: six policy makers at the national level who are heavily involved in health resource allocation decisions in Thailand specifically on HIV/AIDS ('policy makers'); six members of the Thai network for PLWHA, representing PLWHA groups at the regional level in Thailand ('PLWHA'); and six community members who have been trained by public health providers to be the VHVs in Samutprakan province ('VHVs'). Participants were selected purposively from each group of stakeholders on the basis of their participation in the previous DCE study to ensure that they were familiar with the DCE and the priority setting process. Each group discussion began with a brief introduction of the purpose of the meeting. Next, participants were presented with the rank ordering of the interventions, and they were then asked whether they agreed with the rank and to provide their justifications. We then asked them to re-classify all interventions into three categories - based on the traffic-light analogy: 'good candidates for implementation' (green), 'not good candidates for implementation' (red), and 'in-between' (yellow). This re-classification was done through consensus or, when necessary, through voting. In all steps, participants were encouraged by the researcher (SY) to discuss, bring in additional criteria, and share their opinions with justifications regarding their preferences.

### Comparison of rankings

We compared the rank ordering of interventions before and after the deliberative process to explore the impact of deliberation by estimating the Spearman's rank correlation coefficient.

### Research ethics

This study was approved by the Institute for the Development of Human Research Protections, Ministry of Public Health, Thailand. All participants provided their written informed consent.

## Results

The rank ordering of the 40 HIV/AIDS interventions before and upon deliberation is presented in Table 1. As indicated by the ranking results before deliberation, the group of policy makers expressed a preference for preventive programs that are highly effective and target high risk groups such as men who have sex with men (MSM), injecting drug users (IDU), female sex workers (FSW), and HIV sero-discordant couples, with good quality of evidence on intervention effectiveness. The five interventions with the highest priority were voluntary counseling and testing (VCT) for IDU, street outreach for IDU, substitution treatment for IDU, improved sexual transmitted infection (STI) treatment services for IDU, and improved STI treatment services for HIV sero-discordant couples.

**Table 1:** The HIV/AIDS interventions' ranking based on DCE, and the ranking after group discussions

HIV/AIDS intervention (target group)	Ranking*											
	Policy makers				PLWHA				VHVs			
	DCE^† ^			Group discussion^‡^	DCE			Group discussion	DCE			Group discussion
	Probability of selection (%)	(95% CI)	Rank		Probability of selection (%)	(95% CI)	Rank		Probability of selection (%)	(95% CI)	Rank	
Community based education (MSM)	98.82	(94.9-99.7)	11	3	64.34	(45.2-79.8)	15	1	71.28	(48.9-86.6)	15	3
Community based education (IDU)	96.73	(86.1-99.3)	14	3	79.67	(63.1-90.0)	11	1	78.31	(56.8-90.8)	13	3
Community based education (Youth)	99.86	(98.8-100)	2	3	92.14	(79.0-97.3)	2	1	96.56	(87.6-99.1)	1	1
Community based education (FSW)	99.79	(97.8-100)	4	3	78.57	(51.9-92.6)	12	1	90.81	(69.1-97.8)	5	3
Workplace based education ± condom distribution/free STI clinic (FSW)	99.22	(94.4-99.9)	8	1	71.97	(47.4-88.0)	14	1	87.45	(66.1-96.1)	9	2
Workplace based education ± condom distribution/free STI clinic (general public)	98.58	(92.4-99.7)	12	3	84.45	(67.7-93.4)	9	1	87.06	(67.8-95.5)	10	2
Workplace based education ± condom distribution/free STI clinic (male conscripts in military camps)	99.84	(99.0-100)	3	2	77.15	(56.5-90.0)	13	1	89.03	(71.7-96.3)	8	2
School-based sex education programmes (+ life skills)	99.49	(96.8-99.9)	7	1	89.15	(75.8-95.6)	4	1	95.19	(86.0-98.4)	2	1
Peer education (MSM)	98.82	(94.9-99.7)	11	1	64.34	(45.2-79.8)	15	1	71.28	(48.9-86.6)	15	2
Peer education (IDU)	99.10	(94.3-99.9)	9	1	84.84	(67.2-93.9)	8	1	83.66	(60.2-94.5)	11	3
Peer education (Youth)	99.00	(93.9-99.8)	10	3	86.24	(70.5-94.3)	7	1	89.56	(72.8-96.5)	7	1
Peer education (FSW)	99.79	(97.8-100)	4	1	78.57	(51.9-92.6)	12	1	90.81	(69.1-97.8)	5	3
Mass media campaign (general public)	98.58	(92.4-99.7)	12	3	84.45	(67.7-93.4)	9	1	87.06	(67.8-95.5)	10	1
VCT ± STI clinic/Condom distribution (Prison inmate)	99.54	(97.0-99.9)	6	1	88.01	(72.9-95.2)	6	1	92.19	(77.7-97.6)	4	2
VCT ± STI clinic/Condom distribution (MSM)	99.84	(99.0-100)	3	1	77.15	(56.5-89.8)	13	1	89.03	(71.7-96.3)	8	3
VCT ± STI clinic/Condom distribution (IDU)	99.87	(98.8-100)	1	1	91.29	(76.4-97.1)	3	1	94.36	(80.0-98.6)	3	1
VCT ± STI clinic/Condom distribution (HIV sero-discordant couples)	99.54	(97.0-99.9)	6	1	88.01	(72.9-95.2)	6	1	92.19	(77.7-97.6)	4	2
VCT ± STI clinic/Condom distribution (Youth)	99.49	(96.8-99.9)	7	2	89.15	(75.8-95.6)	4	1	95.19	(86.0-98.4)	2	1
VCT ± STI clinic/Condom distribution (FSW)	99.22	(94.4-99.9)	8	1	71.97	(47.3-88.0)	14	1	87.45	(66.1-96.1)	9	3
VCT ± STI clinic/Condom distribution (general public)	99.61	(97.0-100)	5	2	88.57	(71.5-96.0)	5	1	90.51	(70.7-97.4)	6	2
Routine (provider-initiated) voluntary HIV screening at healthcare settings (general public)	99.61	(97.0-100)	5	2	88.57	(71.5-96.0)	5	1	90.51	(70.7-97.4)	6	1
Condom use (availability and accessibility) (FSW)	99.79	(97.8-100)	4	1	78.57	(51.9-92.6)	12	1	90.81	(69.1-97.8)	5	2
Condom use (availability and accessibility) (general public)	98.58	(92.4-99.7)	12	3	84.45	(67.7-93.4)	9	1	87.06	(67.8-95.5)	10	1
Condom use (availability and accessibility) (HIV sero-discordant couples)	99.54	(97.0-99.9)	6	1	88.01	(72.9-95.2)	6	1	92.19	(77.7-97.6)	4	1
Condom use (availability and accessibility) (MSM)	99.84	(99.0-100)	3	1	77.15	(56.5-89.8)	13	1	89.03	(71.7-96.3)	8	2
Street outreach (IDU)	99.87	(98.8-100)	1	1	91.29	(76.4-97.1)	3	1	94.36	(80.0-98.6)	3	1
Substitution treatment (IDU)	99.87	(98.8-100)	1	1	91.29	(76.4-97.1)	3	1	94.36	(80.0-98.6)	3	3
Using nucleic acid test screening (NAT) of voluntary blood donations (general public)	98.58	(92.4-99.7)	12	1	84.45	(67.7-93.4)	9	2	87.06	(67.8-95.5)	10	3
Screening blood products and donated organs for HIV (general public)	98.58	(92.4-99.7)	12	1	84.45	(67.7-93.4)	9	1	87.06	(67.8-95.5)	10	2
Improved STI treatment services (MSM)	99.84	(99.0-100)	3	1	77.15	(56.5-89.8)	13	2	89.03	(71.7-96.3)	8	2
Improved STI treatment services (IDU)	99.87	(98.8-100)	1	1	91.29	(76.4-97.1)	3	2	94.36	(80.0-98.6)	3	1
Improved STI treatment services (HIV sero-discordant couples)	99.87	(98.8-100)	1	1	91.29	(76.4-97.1)	3	2	94.36	(80.0-98.6)	3	2
Improved STI treatment services (Youth)	99.86	(98.8-100)	2	1	92.14	(79.0-97.3)	2	2	96.56	(87.6-99.1)	1	1
Improved STI treatment services (FSW)	99.79	(97.8-100)	4	1	78.57	(51.9-92.6)	12	2	90.81	(69.1-97.8)	5	2
Improved STI treatment services (general public)	99.61	(97.0-100)	5	1	88.57	(71.5-96.0)	5	2	90.51	(70.7-97.4)	6	2
Prevention mother to child transmission	99.79	(97.8-100)	4	1	78.57	(51.9-92.6)	12	1	90.81	(69.1-97.8)	5	2
PEP for healthcare workers	97.25	(86.1-99.5)	13	1	80.55	(61.5-91.5)	10	1	74.46	(47.8-90.3)	14	3
Increase alcohol tax	98.58	(92.4-99.7)	12	3	84.45	(67.7-93.4)	9	3	87.06	(67.8-95.5)	10	3
Highly active antiretroviral therapy for HIV/AIDS	95.64	(89.0-99.8)	15	1	94.92	(89.0-98.7)	1	1	82.24	(55.4-94.5)	12	2
Definitive treatment and care for opportunistic infections, and other palliative care	95.64	(89.0-99.8)	15	1	94.92	(89.0-98.7)	1	1	82.24	(55.4-94.5)	12	2

DCE, discrete choice experiment; PLWHA, people living with HIV/AIDS; VHVs, village health volunteers; MSM, men who have sex with men; IDU, injectable drug users; FSW, female sex workers; STI, sexual transmitted infection; VCT, voluntary counseling and testing; PEP, post-exposure prophylaxis

*Rank 1 is for the interventions in a group of the highest probability of selection comparing to others on the list

^†^The rankings from DCE depend on each group of stakeholders that unable to compare cross the groups

^‡^The rankings from group discussion of each group of stakeholders categorized into three groups through consensus; rank 1 is the intervention that was probable 'good candidate for implementation'; rank 2 is the intervention that was probable 'in between'; and rank 3 is the intervention that was probable 'not good candidate for implementation'

Upon deliberation, the group of policy makers reinforced their preference for highly effective programs that target high risk groups. Community-based education and programs that target the youth or the general population (with the exception for those aimed at the improvement of STI treatment services) were not preferred. In the deliberative process, a number of additional criteria were put forward in addition to those identified in the DCE. The policy maker group proposed cost-effectiveness as an important additional criterion. This group also added the criteria of whether an intervention could be used for multiple purposes, and of safety. For example, a policy maker argued that introducing nucleic acid test screening for blood testing enables the Thai Red Cross Society to simultaneously investigate the existence of Hepatitis B and C with detecting HIV in the same specimen, thus creating added value. Also, a reliable blood donation system is very important to secure safety in Thailand in this respect. The other criterion mentioned was the importance of targeting health care workers at risk as a way of encouraging them to work with PLWHA in hospitals. This led to a change from the rank 10^th ^of the post-exposure prophylaxis for healthcare workers before deliberation to 'good candidate for implementation' category, upon deliberation.

The group of PLWHA expressed a strong preference for the treatment or care for AIDS patients i.e. highly active antiretroviral therapy, and treatment for opportunistic infection and other palliative care, as elicited by the DCE study (Table 1). However, upon deliberation, PLWHA gave almost all of the 40 interventions the same priority. They argued that every intervention was important and should be implemented together to prevent HIV infection. This group of PLWHA also asked that more budget possibilities be found from several sources of funding in order to secure the programs and that otherwise, HIV/AIDS programs should be smaller in scope so policy makers can cover all programs within their limited budgets.

PLWHA suggested the availability of alternatives as an additional criterion. For example, improving STI treatment services was not seen as a priority as alternative services were available in hospitals. PLWHA strongly disagreed with considering cost-effectiveness as a criterion - they argued that if an intervention is effective, it should be implemented, and that financial considerations should not be important. PLWHA also prioritized interventions that target the general population rather than high risk groups, because interventions for the general population cover a larger segment of the population, and reflect their notion that everyone has equal risk of HIV infection. One participant argued: "If these (interventions) are the national policy, they should be implemented to everyone not only the high risk groups. This is because everyone is at equal risk of HIV infection. We are all the same".

The preferences of VHVs cohered largely with those of policy makers except for the target group of the interventions: VHVs preferred interventions that target the youth rather than high risk populations. Consequently, community-based education and improvement of STI treatment services for the youth were the highest priority (Table 1). This preference was also confirmed upon deliberation. VHVs introduced the number of beneficiaries as an additional criterion. One volunteer mentioned: "Mass media campaigns have an impact on lots of people in society. So we think this intervention is beneficial for society at large". Furthermore, VHVs emphasized the need to adapt certain interventions to suit the groups targeted.

There was a significant correlation between the rank ordering before and after deliberation for policy makers (correlation coefficient 37%) and VHVs (46%). The correlation coefficient presents the consistency of results between the DCE ranking and deliberation ranking. No such significant correlation was found for the PLWHA. In addition, from the group discussions, we found that both policy makers and VHVs were generally positive about the ease of interpreting DCE results and the MCDA process, whereas PLWHA were generally negative because of the difficulty of the DCE questionnaire, which might lead to a misunderstanding of the exercise among the respondents.

## Discussion

This study has experimented with the use of MCDA to guide the priority setting of HIV/AIDS interventions in Thailand, on the basis of consultations with the relevant stakeholders through a deliberative process.

This study revealed the importance of five criteria included in the DCE (i.e. target groups of interventions, gender of target groups, type of interventions, effectiveness, and quality of evidence on effectiveness), and a number of additional criteria raised during the deliberative process (i.e. ethical and social concerns, cost-effectiveness, (non)availability of alternatives; number of beneficiaries; and inappropriate use or abuse of interventions). This reflects that stakeholders consider multiple criteria in prioritising interventions.

The abovementioned results highlight that MCDA has good potential to be used for the making of explicit prioritisation decisions. Also, we observed that the group of policy makers and VHVs - although not PLWHA respondents - applauded the systematic approach for priority setting, including the development of relevant criteria, the presentation of the performance of interventions against these criteria, and the deliberative process. Although MCDA seems difficult for PLWHA as they may not be familiar or comfortable to make trade-off decisions, the considerable overlap of the rank ordering before and upon deliberation in the group of policy makers and VHVs indicates that the quantifiable criteria used in the DCE partly reflect the concerns that stakeholders have in their intervention priorities. We believe that, through its explicit approach, MCDA contributes to the transparency and accountability of the priority setting process. Moreover, the provision of the DCE ranking reduces the stream of information that stakeholders need to absorb when prioritising many interventions simultaneously. We therefore advocate that the identification and weighing of quantifiable criteria (whether through DCE or any other technique) should also be considered as an integrated MCDA component.

The present application of MCDA seems especially useful for policy planning in the long run as it can set priorities among a large set of interventions without defining the allocation of resources in a precise fashion. This use, also labeled *generalized priority setting*, can have far-reaching and constructive influences on policy formulation in the long term [26]. In contrast, the use of MCDA as presented in this study may not be useful for guiding highly contextualized decisions on the implementation of a single intervention, since this requires a higher level of detail in terms of financial and budgeting considerations.

This study has experimented with the inclusion of a process of deliberation in MCDA in a research environment. As of now, Thailand is stepping towards a routine application of MCDA to define its universal coverage benefit package. Observations of that process reveal that the inclusion of all relevant stakeholders right from the beginning of the MCDA process is imperative to its success [29].

Yet, we also observed a number of shortcomings in the use of MCDA in this study. First, DCE are cognitive demanding and may not be appropriate for all stakeholders. Most notably, PLWHA had difficulties in completing the DCE survey and interpreting the DCE findings. Further research is needed on the use of less cognitive demanding techniques than DCE that serve the same goal [30]. Second, our intervention set was relatively homogeneous in terms of the criteria covered in the DCE (e.g. effectiveness; quality of evidence on effectiveness; type of intervention), and this resulted in low variation in the probabilities of inclusion. The application of DCE across different health conditions [15-19] is, in that respect, more powerful. Third, we did not engage all stakeholders in a single deliberative process to arrive at a consensus on the rank ordering of interventions, an adaption which would represent the final stage of a successful priority setting process. However, the findings in this study can serve as a reflection of other stakeholders' preferences for policy decision making that may lead to greater acceptance of priority setting decisions. Moreover, this study can be considered a lesson learned process for other stakeholders, especially the general population who have never been involved in health policy decision-making, and can help them to understand how to set priorities for health interventions. In future priority setting research, it would therefore be valuable to incorporate these public perspectives.

Although the set of criteria for MCDA may vary by country and health system context, the approach is generalizable to other settings. Furthermore, the MCDA criteria may be different if priority setting is required across different health problems e.g. infectious diseases, cardiovascular conditions, and mental health problems. Therefore, further exploration is warranted.

## Conclusion

This study has documented the feasibility of MCDA for prioritising HIV/AIDS interventions in Thailand, and has shown the usefulness of a deliberative process as an integrated component of MCDA. MCDA holds potential to contribute to a more transparent and accountable priority setting process, and further application of this approach in the prioritisation of health interventions is warranted.

## Competing interests

The authors declare that they have no competing interests.

## Authors' contributions

SY, RB, and YT conceptualized the study. SY wrote the first draft of the manuscript. RB, ST, and YT made substantial contributions to the data interpretation and writing of the manuscript. All authors read and approved the final manuscript.

## Supplementary Material

Additional file 1 **Appendix 1**. The performance matrix of HIV/AIDS interventions. **Appendix 2**. Discrete choice model results by perspective. Click here for file

## References

[B1] UNAIDS/WHO working on global HIV/AIDS and STI Epidemiological fact sheet on HIV and AIDS: Core data on epidemiology and response 2008 Geneva: World Health Organization

[B2] Porapakkham Y Rao C Pattaraarchachai J Polprasert W Vos T Adair T Lopez AD Estimated causes of death in Thailand, 2005: implications for health policy Population Health Metrics 2010 8 14 10.1186/1478-7954-8-14 20482761 PMC2885317

[B3] Tantivess S Universal access to antiretroviral therapy in Thailand: an analysis of the policy process 2006 University of London: PhD thesis

[B4] Pattanaphesaj J Teerawattananon Y Reviewing the evidence on effectiveness and cost-effectiveness of HIV prevention strategies in Thailand BMC Publ Health 2010 10 401 10.1186/1471-2458-10-401 PMC2912810 20604975

[B5] Hogan DR Baltussen R Hayashi C Lauer J Salomon JA Cost effectiveness analysis of strategies to combat HIV/AIDS in developing countries BMJ 2005 331 7530 1431 1437 10.1136/bmj.38643.368692.68 16282380 PMC1315644

[B6] Creese A Floyd K Alban A Guinness L Cost-effectiveness of HIV/AIDS interventions in Africa: a systematic review of the evidence Lancet 2002 359 1635 1642 10.1016/S0140-6736(02)08595-1 12020523

[B7] Cohen DA Wu S-Y Farley TA Cost-effectiveness allocation of government funds to prevent HIV infection Heal Aff 2005 24 915 926 10.1377/hlthaff.24.4.915 16136633

[B8] Kaplan EH Merson MH Allocating HIV-prevention resources: Balancing efficiency and equity Am J Public Health 2002 92 12 1905 1907 10.2105/AJPH.92.12.1905 12453805 PMC1447350

[B9] Kenny N Joffres C An ethical analysis of international health priority-setting Health Care Anal 2008 16 145 160 10.1007/s10728-007-0065-5 18449806

[B10] Tantivess S Walt G Using cost-effectiveness analyses to inform policy: the case of antiretroviral therapy in Thailand Cost Effectiveness and Resource Allocation 2006 4 21 10.1186/1478-7547-4-21 17196110 PMC1779364

[B11] Musgrove P Public spending on health care: how are different criteria related? Health Policy 1999 47 207 223 10.1016/S0168-8510(99)00024-X 10538919

[B12] Dolan P Shaw R A note on the relative importance that people attach to different factors when setting priorities in health care Health Expectation 2002 6 53 59 10.1046/j.1369-6513.2003.00210.x PMC5060165 12603628

[B13] Multi-criteria analysis manual http://iatools.jrc.ec.europa.eu/public/IQTool/MCA/DTLR_MCA_manual.pdf

[B14] Baltussen R Niessen LW Priority setting of health interventions: the need for multi-critieria decision analysis Cost Effectiveness and Resource Allocation 2006 4 14 10.1186/1478-7547-4-14 16923181 PMC1560167

[B15] Baltussen R Stolk E Chisholm D Aikins M Towards a multi-criteria approach for priority setting: an application to Ghana Heal Econ 2006 15 689 696 10.1002/hec.1092 16491464

[B16] Baltussen R ten Asbroek A Koolman X Shrestha N Bhattarai P Niessen LW Priority setting using multiple criteria: should a lung health programme be implemented in Nepal? Health Policy and Planning 2007 22 178 185 10.1093/heapol/czm010 17412742

[B17] Green C Gerard K Exploring the social value of health-care interventions: a stated preference discrete choice experiment Heal Econ 2009 18 951 976 10.1002/hec.1414 19034951

[B18] Jehu-Appiah C Baltussen R Acquah C Aikins M d'Almeida SA Bosu WK Koolman X Lauer J Osei D Adjei S Balancing equity and efficiency in health priorities in Ghana: the use of multicriteria decision analysis Value in Health 2009 11 1081 1087 10.1111/j.1524-4733.2008.00392.x 19602214

[B19] Koopmanschap MA Stolk EA Koolman X Dear policy maker: Have you made up your mind? A discrete choice experiment among policy makers and other health professionals Int J Technol Assess Health Care 2010 26 2 198 204 10.1017/S0266462310000048 20392324

[B20] Goetghebeur MM Wagner M Khoury H Rindress D Grégoire J-P Deal C Combining multicriteria decision analysis, ethics and health technology assessment: applying the EVIDEM decisionmking framework to growth hormone for Turner syndrome patients Cost Effectiveness and Resource Allocation 2010 8 4 10.1186/1478-7547-8-4 20377888 PMC2856527

[B21] Culyer AJ Equity of what in healthcare? Why the traditional answers don't help policy- and what to do in the future Healthcare Papers 2007 8 Spec No 12 26 19096262 10.12927/hcpap.2007.19216

[B22] Brock D Wikler D Jamison DT, Breman JG, Measham AR, Alleyne G, Claeson M, Evans DB, Jha P, Mills A, Musgrove P Ethical issues in resource allocation, research, and new products development Disease control priorities in developing countries 2006 2 New York: Oxford University Press and the World Bank

[B23] Kenny N Joffres C An ethical analysis of international health priority setting Health Care Analysis 2008 16 145 160 10.1007/s10728-007-0065-5 18449806

[B24] Kapiriri L Norheim OF Martin DK Fairness and accountability for reasonableness. Do the views of priority setting decision makers differ across health systems and levels of decision making? Social Science & Medicine 2009 68 766 773 10.1016/j.socscimed.2008.11.011 19070414

[B25] Berney L Kelly M Doyal L Feder G Griffiths C Jones IR Ethical principles and the rationing of health care: a qualitative study in general practice Br J Gen Prac 2005 55 620 625 PMC1463218 16105371

[B26] Baltussen R Youngkong S Paolucci F Niessen LW Multi-criteria decision analysis to prioritize health interventions: Capitalizing on first experiences Health Policy 2010 96 262 264 10.1016/j.healthpol.2010.01.009 20206403

[B27] Johri M Norheim OF Can cost-effectiveness analysis integrate equity concerns? A systematic review of current approaches In Paper presented at ISPOR 12th Annual European Congress Paris 2009

[B28] Youngkong S Baltussen R Tantivess S Koolman X Teerawattananon Y Criteria for priority setting of HIV/AIDS interventions in Thailand: A discrete choice experiment BMC Health Service Research 2010 10 197 10.1186/1472-6963-10-197 PMC2912896 20609244

[B29] The Intervention Health Policy Program Health Intervention and Technology Assessment Program Research for development of health benefit package under universal health care coverage scheme 2011 1 Nonthaburi

[B30] Dolan JG Multi-criteria clinical decision support: A primer on the use of multiple criteria decision making methods to promote evidence-based, patient-centered healthcare Patient 2010 3 4 229 248 21394218 10.2165/11539470-000000000-00000 PMC3049911

