# Supplementary material for: Multi-criteria decision analysis for setting priorities on HIV/AIDS interventions in Thailand
Source: Health Res Policy Syst. 2012 Feb 17;10:6. doi: 10.1186/1478-4505-10-6 (PMC3310796; doi:10.1186/1478-4505-10-6)
Supplement: Additional file 1 — Appendix 1. The performance matrix of HIV/AIDS interventions. Appendix 2. Discrete choice model results by perspective. [file 1478-4505-10-6-S1.DOC]

Appendix 1 The performance matrix of HIV/AIDS interventions

| Interventions | Performance of HIV/AIDS interventions on each criterion^†^ | | | | | | | | | | | | | |
| --- | --- | --- | --- | --- | --- | --- | --- | --- | --- | --- | --- | --- | --- | --- |
|  | Target group of intervention | | | | Gender of target group | | | Type of intervention | | | Effectiveness | | Quality of evidence on effectiveness | |
|  | Children | Teenagers | High risk adults | All adults | Male | Female | Both genders | HIV | AIDS | Prevention | Low effective | High effective | weak quality of evidence | Strong quality of evidence |
| Community based education (MSM) | 0 | 0 | 1 | 0 | 1 | 0 | 0 | 0 | 0 | 1 | 1 | 0 | 0 | 1 |
| Community based education (IDU) | 0 | 0 | 1 | 0 | 0 | 0 | 1 | 0 | 0 | 1 | 1 | 0 | 1 | 0 |
| Community based education (Youth) | 0 | 1 | 0 | 0 | 0 | 0 | 1 | 0 | 0 | 1 | 0 | 1 | 0 | 1 |
| Community based education (FSW) | 0 | 0 | 1 | 0 | 0 | 1 | 0 | 0 | 0 | 1 | 0 | 1 | 0 | 1 |
| Workplace based education ± condom/free STI clinic (FSW) | 0 | 0 | 1 | 0 | 0 | 1 | 0 | 0 | 0 | 1 | 0 | 1 | 1 | 0 |
| Workplace based education ± condom/free STI clinic (general public) | 0 | 0 | 0 | 1 | 0 | 0 | 1 | 0 | 0 | 1 | 0 | 1 | 1 | 0 |
| Workplace based education ± condom/free STI clinic (male conscripts in military camps) | 0 | 0 | 1 | 0 | 1 | 0 | 0 | 0 | 0 | 1 | 0 | 1 | 0 | 1 |
| School-based sex education programmes (+ life skills) (Youth) | 0 | 1 | 0 | 0 | 0 | 0 | 1 | 0 | 0 | 1 | 0 | 1 | 1 | 0 |
| Peer education (MSM) | 0 | 0 | 1 | 0 | 1 | 0 | 0 | 0 | 0 | 1 | 1 | 0 | 0 | 1 |
| Peer education (IDU) | 0 | 0 | 1 | 0 | 0 | 0 | 1 | 0 | 0 | 1 | 1 | 0 | 0 | 1 |
| Peer education (Youth) | 0 | 1 | 0 | 0 | 0 | 0 | 1 | 0 | 0 | 1 | 1 | 0 | 0 | 1 |
| Peer education (FSW) | 0 | 0 | 1 | 0 | 0 | 1 | 0 | 0 | 0 | 1 | 0 | 1 | 0 | 1 |
| Mass media campaign (General Public) | 0 | 0 | 0 | 1 | 0 | 0 | 1 | 0 | 0 | 1 | 0 | 1 | 1 | 0 |
| VCT + STI clinic/condom distribution (Prison inmate) | 0 | 0 | 1 | 0 | 0 | 0 | 1 | 0 | 0 | 1 | 0 | 1 | 1 | 0 |
| VCT + STI clinic/condom distribution (HIV sero-discordant couples) | 0 | 0 | 1 | 0 | 0 | 0 | 1 | 0 | 0 | 1 | 0 | 1 | 1 | 0 |
| VCT + STI clinic/condom distribution (MSM) | 0 | 0 | 1 | 0 | 1 | 0 | 0 | 0 | 0 | 1 | 0 | 1 | 0 | 1 |
| VCT + STI clinic/condom distribution (IDU) | 0 | 0 | 1 | 0 | 0 | 0 | 1 | 0 | 0 | 1 | 0 | 1 | 0 | 1 |
| VCT + STI clinic/condom distribution (Youth) | 0 | 1 | 0 | 0 | 0 | 0 | 1 | 0 | 0 | 1 | 0 | 1 | 1 | 0 |
| VCT + STI clinic/condom distribution (FSW) | 0 | 0 | 1 | 0 | 0 | 1 | 0 | 0 | 0 | 1 | 0 | 1 | 1 | 0 |
| VCT + STI clinic/condom distribution (General Public) | 0 | 0 | 0 | 1 | 0 | 0 | 1 | 0 | 0 | 1 | 0 | 1 | 0 | 1 |
| Routine (provider-initiated) voluntary HIV screening at healthcare settings (General Public) | 0 | 0 | 0 | 1 | 0 | 0 | 1 | 0 | 0 | 1 | 0 | 1 | 0 | 1 |
| Condom use (availability and accessibility) (MSM) | 0 | 0 | 1 | 0 | 1 | 0 | 0 | 0 | 0 | 1 | 0 | 1 | 0 | 1 |
| Condom use (availability and accessibility) (FSW) | 0 | 0 | 1 | 0 | 0 | 1 | 0 | 0 | 0 | 1 | 0 | 1 | 0 | 1 |
| Condom use (availability and accessibility) (General Public) | 0 | 0 | 0 | 1 | 0 | 0 | 1 | 0 | 0 | 1 | 0 | 1 | 1 | 0 |
| Condom use (availability and accessibility) (HIV sero-discordant couples) | 0 | 0 | 1 | 0 | 0 | 0 | 1 | 0 | 0 | 1 | 0 | 1 | 1 | 0 |
| Street outreach (IDU) | 0 | 0 | 1 | 0 | 0 | 0 | 1 | 0 | 0 | 1 | 0 | 1 | 0 | 1 |
| Substitution treatment (IDU) | 0 | 0 | 1 | 0 | 0 | 0 | 1 | 0 | 0 | 1 | 0 | 1 | 0 | 1 |
| Using nucleic acid test screening (NAT) of voluntary blood donations (General Public) | 0 | 0 | 0 | 1 | 0 | 0 | 1 | 0 | 0 | 1 | 0 | 1 | 1 | 0 |
| Screening blood products and donated organs for HIV (General Public) | 0 | 0 | 0 | 1 | 0 | 0 | 1 | 0 | 0 | 1 | 0 | 1 | 1 | 0 |
| Improved STI treatment servcies (MSM) | 0 | 0 | 1 | 0 | 1 | 0 | 0 | 0 | 0 | 1 | 0 | 1 | 0 | 1 |
| Improved STI treatment services (IDU) | 0 | 0 | 1 | 0 | 0 | 0 | 1 | 0 | 0 | 1 | 0 | 1 | 0 | 1 |
| Improved STI treatment services (HIV sero-discordant couples) | 0 | 0 | 1 | 0 | 0 | 0 | 1 | 0 | 0 | 1 | 0 | 1 | 0 | 1 |
| Improved STI treatment services (Youth) | 0 | 1 | 0 | 0 | 0 | 0 | 1 | 0 | 0 | 1 | 0 | 1 | 0 | 1 |
| Improved STI treatment services (FSW) | 0 | 0 | 1 | 0 | 0 | 1 | 0 | 0 | 0 | 1 | 0 | 1 | 0 | 1 |
| Improved STI treatment services (General Public) | 0 | 0 | 0 | 1 | 0 | 0 | 1 | 0 | 0 | 1 | 0 | 1 | 0 | 1 |
| Prevention mother to child transmission | 0 | 0 | 1 | 0 | 0 | 1 | 0 | 0 | 0 | 1 | 0 | 1 | 0 | 1 |
| PEP for healthcare workers | 0 | 0 | 0 | 1 | 0 | 0 | 1 | 0 | 0 | 1 | 1 | 0 | 0 | 1 |
| Increased alcohol tax | 0 | 0 | 0 | 1 | 0 | 0 | 1 | 0 | 0 | 1 | 0 | 1 | 1 | 0 |
| Highly active antiretroviral therapy for AIDS patients | 0 | 0 | 0 | 1 | 0 | 0 | 1 | 0 | 1 | 0 | 0 | 1 | 0 | 1 |
| Highly active antiretroviral therapy for HIV infection | 0 | 0 | 0 | 1 | 0 | 0 | 1 | 1 | 0 | 0 | 0 | 1 | 0 | 1 |
| Definitive treatment and care for opportunistic infections, and other palliative care | 0 | 0 | 1 | 0 | 0 | 0 | 1 | 0 | 1 | 0 | 0 | 1 | 0 | 1 |

DCE, discrete choice experiment; PLWHA, people living with HIV/AIDS; VHVs, village health volunteers; MSM, men who have sex with men; IDU, injectable drug users; FSW, female sex workers; STI, sexual transmitted infection; VCT, voluntary counseling and testing; PEP, post-exposure prophylaxis

^†^ ‘0’ denotes the absence, and ‘1’ denotes the presence.

Appendix 2 Discrete choice model results by perspective†

|  | |  | Perspectives | | | | | |
| --- | --- | --- | --- | --- | --- | --- | --- | --- |
|  |  |  | Policy makers | | People living with HIV/AIDS | | Village Health Volunteers | |
| Criteria | Levels |  | Coefficient  (95% CI) | (p-value) | Coefficient  (95% CI) | (p-value) | Coefficient  (95% CI) | (p-value) |
|  |  |  |  |  |  |  |  |  |
| Target group | Child |  |  |  |  |  |  |  |
|  | Teen |  | 1.049* | (0.001) | 0.135 | (0.385) | 0.830* | (0.000) |
|  |  |  | (0.445, 1.654) | | (-0.169, 0.440) | | (0.464, 1.196) | |
|  | HiRrisk |  | 1.153* | (0.001) | 0.022 | (0.900) | 0.314 | (0.142) |
|  |  |  | (0.502, 1.803) | | (-0.323, 0.368) | | (-0.105, 0.734) | |
|  | Adults |  | 0.023 | (0.926) | -0.279 | (0.065) | -0.249 | (0.176) |
|  |  |  | (-0.470, 0.517) | | (-0.575, 0.017) | | (-0.609, 0.112) | |
| Gender of target group | Male |  |  |  |  |  |  |  |
|  | Female |  | -0.256 | (0.321) | 0.082 | (0.544) | 0.196 | (0.229) |
|  |  |  | (-0.762, 0.250) | | (-0.184, 0.348) | | (-0.123, 0.514) | |
|  | BothGen | | 0.266 | (0.189) | 1.132* | (0.000) | 0.724* | (0.000) |
|  |  | | (-0.131, 0.663) | | (0.911, 1.354) | | (0.458, 0.990) | |
| Type of intervention | HIV |  |  |  |  |  |  |  |
|  | AIDS |  | -0.493* | (0.019) | 1.091* | (0.000) | -0.476* | (0.001) |
|  |  |  | (-0.904, -0.081) | | (0.869, 1.313) | | (-0.744, -0.208) | |
|  | Prevent | | 1.967* | (0.000) | 0.212 | (0.116) | 0.246 | (0.137) |
|  |  | | (1.450, 2.485) | | (-0.052, 0.476) | | (-0.078, 0.569) | |
| Effectiveness | LoEff |  |  |  |  |  |  |  |
|  | HiEff |  | 1.983* | (0.000) | 0.627* | (0.000) | 1.185* | (0.000) |
|  |  |  | (1.643, 2.323) | | (0.454, 0.800) | | (0.973, 1.395) | |
| Quality of evidence | Weak |  |  |  |  |  |  |  |
| on effectiveness | Strong |  | 1.310* | (0.000) | 0.356* | (0.000) | 0.349* | (0.001) |
|  |  |  | (0.976, 1.645) | | (0.183, 0.528) | | (0.139, 0.560) | |
|  |  |  |  |  |  |  |  |  |
| Log likelihood | |  | -424.4532 | | -1434.3323 | | -963.3818 | |
| Pseudo R^2^ | |  | 0.2747 | | 0.0992 | | 0.0984 | |
| Hosmer-Lemeshow chi-square  (p-value) | |  | 1.36  (0.995) | | 2.79  (0.947) | | 1.87  (0.985) | |

*Significant variables (p < 0.05)

^†^Source: Youngkong S, Baltussen R, Tantivess S, Koolman X, Teerawattananon Y: Criteria for priority setting of HIV/AIDS interventions in Thailand: A discrete choice experiment. BMC Health Service Research 2010, 10:197.
